# The correlation between non-suicidal self-injury in adolescents, parenting styles, and borderline personality traits

**DOI:** 10.3389/fpsyt.2025.1620872

**Published:** 2025-08-20

**Authors:** Yuanyuan Dai, Ping Yang, Wensai Ji, Liang Diao, Lu Han

**Affiliations:** ^1^ Wuhu Fourth People's Hospital, Wuhu, China; ^2^ Suzhou Guangji Hospital, The Affiliated Guangji Hospital of Soochow University, Suzhou, China; ^3^ No.971 Hospital Of The People’s Liberation Army Navy, Qingdao, China; ^4^ Qingdao Shinan District Education Support Center, Qingdao, China; ^5^ Qingdao Municipal Hospital, University of Health and Rehabilitation Sciences, Qingdao, China

**Keywords:** non-suicidal self-injury (NSSI), parental styles, adolescents, borderline personality traits, mental health, depressive episode

## Abstract

**Introduction:**

This study aimed to investigate the associations between parenting styles, borderline personality traits, and non-suicidal self-injury (NSSI) in adolescents with depression, thereby providing a theoretical foundation for targeted psychological interventions.

**Methods:**

This study included 101 adolescent patients with depressive episodes admitted to the Fourth People’s Hospital of Wuhu City, Anhui Province, from May 2022 to October 2023 (23 males and 78females). Two attending physicians or those of higher ranks diagnosed these patients as having depressive episodes based on the Diagnostic and Statistical Manual of Mental Disorders, Fifth Edition (DSM-5).Data were collected using the Adolescent Non-Suicidal Self-Injury Behavior Assessment Questionnaire, the Parental Rearing Style Evaluation Scale, and relevant items from the Borderline Personality Questionnaire for survey administration.

**Results:**

The frequent NSSI group showed significantly higher scores than the non-frequent group in paternal rejection/denial, maternal overprotection/interference, and total borderline personality traits (all P<0.01, Cohen’s d>0.2). Additionally, the frequent NSSI group exhibited greater paternal overprotection and maternal rejection/denial (both P<0.05, Cohen’s d>0.2). Significant positive correlations were observed between NSSI scores and paternal rejection/denial, maternal overprotection/interference, maternal rejection/denial, and borderline personality traits (all P<0.01), while paternal overprotection was positively correlated with NSSI scores (P<0.05). Multivariate analysis identified paternal rejection/denial, maternal overprotection/interference, and borderline personality traits as independent risk factors for NSSI behaviors (all P<0.05).

**Conclusion:**

These findings suggest that paternal rejection and denial, maternal overprotection and interference, and borderline personality traits may be associated with adolescent NSSI behaviors. The results provide valuable insights for clinicians to better understand the psychosocial contributing factors of NSSI, highlighting that future intervention strategies should consider the combined effects of family parenting patterns and adolescents’ personality characteristics. This study employed a cross - sectional design, which rendered it impossible to establish the multiple causal relationships among parental parenting styles, borderline personality traits, and non - suicidal self - injury (NSSI) behaviors, as well as to analyze the mediating effects between these variables. Future research could integrate multiple research methodologies, such as combining cross - sectional and longitudinal studies.

## Introduction

Non-suicidal self-injury (NSSI) refers to a deliberate act of self-harm that is not intended to result in death, involving the direct and intentional damage to one’s own body tissue, which is not socially accepted (1). Common methods of self-injury include cutting the skin, burning with hot objects, pulling hair forcefully, or violently hitting the body (2). Given the unique clinical characteristics and the harm associated with NSSI, the DSM-5 has included it in its appendix, emphasizing the need for further research. This inclusion suggests that NSSI may potentially be recognized as an independent mental disorder in the future (3). NSSI among adolescents remains a significant public health issue worldwide. A recent systematic review and meta-analysis found that the global prevalence of NSSI among adolescents aged 12–18 is 17.2%, with a higher incidence among females (19.7%) than males (14.8%) (4). Research has found that although the ultimate goal of NSSI is not suicide, the risk of suicide in this population is much higher than in the general population, making it a significant risk factor for suicidal behavior (5).

Depressive disorder, a prevalent chronic psychiatric condition characterized by persistent low mood, diminished interest, and anhedonia as core symptoms, can occur across all age groups (6). According to the ≪China National Mental Health Development Report (2019-2020)≫, the detection rate of depressive disorder among Chinese adolescents reaches 24.6%. Substantial evidence has demonstrated a strong association between NSSI and depressive disorder, with NSSI being highly prevalent among adolescents with depressive symptoms (7–9). In recent years, China has witnessed a concerning rise in both NSSI behaviors and depressive disorder incidence among adolescents (10, 11), posing significant threats to their physical and psychological well-being.

The risk factors associated with Non-Suicidal Self-Injury (NSSI) among adolescents are complex and multifaceted. Research suggests that NSSI is not caused by a single factor but rather results from a combination of environmental influences, such as parenting styles, and neurobiological factors related to genetics. Currently, the study of risk factors for non-suicidal self-injurious behavior is still in its nascent stages. Relevant studies have revealed that self-injurious behaviors among college students are closely linked to their developmental processes, relationships with parents, family environment, coping strategies for challenges, social relationships, and personality traits (12–15). Studies have identified female gender, negative parenting styles, childhood trauma, and borderline personality traits as significant predictors of non-suicidal self-injury (NSSI) in adolescents (16, 17).

The incidence of non - suicidal self - injury (NSSI) is notably elevated among adolescents with depression. Existing research has indicated that parental parenting styles (e.g., rejection, overprotection) and borderline personality traits are significant predictors of NSSI (18–21). However, the majority of studies have only focused on a single factor or centered on the general adolescent population, failing to uncover the interactive effects of parenting styles and borderline personality traits in depressed patients (22, 23). Furthermore, NSSI in depressed adolescents may be more strongly driven by emotions, and borderline personality traits might further undermine their ability to cope with family – related stress. Yet, this underlying mechanism remains unclear (19). This study focuses on the specific group of Chinese adolescents experiencing depressive episodes, breaking through the limitations of most previous research that solely concentrated on general adolescents or single – diagnosis clinical samples. By including patients diagnosed with depression according to the DSM – 5 criteria, this study more precisely uncovers the unique pathogenesis of non – suicidal self – injury (NSSI) under comorbid conditions. It fills the research gap in this particular sub – population.

Given that non–suicidal self–injury (NSSI) behavior is frequently reported as a strategy for coping with intense negative emotions, the emotion regulation theory (24) provides a critical perspective for this study. This theory underscores the variability in individuals’ ability to exert influence during the emotion generation process. Maladaptive parenting environments (e.g., emotional neglect, overcontrol) may impair adolescents’ capacity to develop adaptive emotion regulation strategies (e.g., acceptance, cognitive reappraisal), compelling them to rely on immediate yet harmful strategies (e.g., NSSI). The core component of borderline personality traits—severe emotional dysregulation—manifests precisely as such deficits in regulatory capacity. Based on this theoretical framework, the present study will explore how parental parenting styles increase the risk of NSSI in adolescents (particularly those with a propensity toward borderline personality traits) by influencing their emotion regulation abilities.

The objective of this study is to investigate the current status of non–suicidal self–injury (NSSI) among adolescents with depressive episode, while conducting an in–depth analysis of the relationship between parental rearing styles and borderline personality traits with NSSI behaviors in this population. Based on existing emotion regulation theories, we propose the following specific research hypotheses: There is a significant positive correlation between the frequency and severity of non–suicidal self–injury (NSSI) behaviors in adolescents and their perceived negative parental parenting styles (such as overprotection, rejection and denial, and harsh punishment). Conversely, there is a significant negative correlation between the frequency and severity of NSSI behaviors and their perceived positive parental parenting styles (such as emotional warmth, understanding, and support). Additionally, there is a significant positive correlation between the level of borderline personality traits in adolescents and the frequency and severity of their NSSI behaviors. This study provides an important reference basis for predicting and preventing non–suicidal self–injury behaviors in adolescents experiencing depressive episode of mood disorders.

## Methods

### Participants

From May 2022 to October 2023, the study subjects were adolescents diagnosed with depressive episode who sought treatment at the Fourth People’s Hospital of Wuhu in Anhui Province.. Based on the sample size calculation formula for case–control studies, *N = Z* ^2^ *× σ* ^2^ */d* ^2^, where *Z* represents the statistical variable (with a confidence level of 95%, *Z*=1.96), *d* denotes the range of sampling error (accounting for a 10% sample attrition rate in this trial), and *σ* is the standard deviation (set at 0.5). The calculation yield*s* n=1.96_2_x0.5_2_/ (10%)_2_=96

. The total sample size for this trial exceeded 96 cases. After screening, clinical interviews were conducted and questionnaires were collected from 102 eligible adolescent patients. However, one case was excluded due to incomplete data, resulting in a final study sample of 101patients. Among them, there were 23males (22.8%) and 78females (77.2%).

Inclusion criteria: (1) Diagnosis of depressive episode according to the Diagnostic and Statistical Manual of Mental Disorders (5th Edition) (DSM–5) by two attending physicians or above, (2) Age range of 13–18 years, (3) Patients with adequate reading ability and good cooperation, capable of completing the scale assessment, (4) Voluntary participation in the study after being informed of the research purpose, with signed informed consent from the participant or their legal guardian.

Exclusion criteria: (1) Patients with comorbid severe mental illness, serious neurological diseases, or severe physical illnesses, (2) Patients with severe language or hearing impairments, (3) Patients who were uncooperative during the psychiatric examination or refused to participate in the study.

This study received approval from the hospital’s Institutional Review Board (IRB) (Approval Number (2021):–KY–06). Given the sensitivity of research involving adolescent patients with depression and related conditions such as non – suicidal self – injury (NSSI) and borderline personality disorder (BPD), the ethical review focused on the following key aspects: the voluntariness of informed consent, psychological protection during scale administration, anonymous data handling, and non – stigmatizing dissemination of research findings (see the ethical approval document for details).

Specific measures were implemented as follows:

1. The research purpose was individually explained to both patients and their guardians, with an emphasis that “participation or non – participation would not affect the treatment.”
2. Immediate psychological support was provided to participants after scale assessment.
3. Data were stored using double – anonymous coding, and the original documents were sealed and kept securely.
4. Research conclusions were presented in terms of group associations to avoid individual labeling (e.g., specific scale scores were not fed back to patients).
5. All participants provided written informed consent.

## Measures

### General information questionnaire

A self–designed questionnaire was utilized to collect demographic information from adolescent patients with depression, including gender, age, grade level, only–child status, satisfaction with academic performance, residence (urban or rural), family income, family structure, and experiences of school bullying. These data were gathered to gain a comprehensive understanding of the patients’ socioeconomic background.

### NSSI assessment questionnaire

The assessment of non – suicidal self – injury (NSSI) was conducted based on the Adolescent NSSI Assessment Questionnaire designed by Wan Yuhui et al. (25). This questionnaire consists of two sub – questionnaires: the Behavior Questionnaire and the Function Questionnaire. The Behavior Questionnaire contains 12 items and is divided into two dimensions: ① Non – severe tissue damage, which refers to behaviors that do not cause obvious and serious physical tissue damage, such as pinching, scratching, and pulling hair, ② Severe tissue damage, which may result in significant bleeding, scratches, and other tissue damage, such as cutting, burning, and carving words or symbols on the skin. The Function Questionnaire comprises 19 items and is categorized into three dimensions: egoistic social interaction, self – negative reinforcement, and emotional expression. Each item is rated on a 5 – point scale ranging from 1 (completely inconsistent) to 5 (completely consistent).The Cronbach’s α coefficient of the Behavior Questionnaire was 0.921, and that of the total score of the Function Questionnaire was 0.905. The questionnaire demonstrated good reliability, and the internal consistency of each sub – dimension was acceptable. Based on the occurrence and frequency of NSSI behaviors over the past year, participants were divided into two groups: those who engaged in NSSI behaviors three or more times were classified into the study group (i.e., the frequent NSSI group), while those who did not engage in NSSI or engaged in it fewer than three times were classified into the control group (i.e., the non–frequent NSSI group). The study group comprised 52 participants, and the control group included 49 participants.

### Suicide questionnaire

The Suicide Questionnaire utilized in this study consisted of three culturally adapted and simplified items designed to evaluate suicidal ideation, suicide plans, and actual suicide attempts among adolescents (26). Participants were required to respond with a clear “yes” or “no” to each of the following questions: “Have you ever seriously considered committing suicide or killing yourself?”, “Have you ever formulated a detailed plan to commit suicide?”, and “Have you ever actually attempted to kill yourself in your lifetime?”

### Parenting style evaluation scale

The Egna Minnen av Barndoms Uppfostran (EMBU) (27) is a psychometric instrument designed to assess individuals’ perceptions of parental rearing behaviors. Originally developed by Perris et al. at Umea University, Sweden, in 1980, the EMBU was subsequently adapted into a Chinese version by Yue Dongmei et al. (27) in 1993 to better align with the cultural context of China. The scale comprises two subscales, one for paternal and one for maternal rearing behaviors, and employs a four–point Likert scale ranging from “never” to “always.” The mean score for each dimension is calculated by summing the item scores and dividing by the number of items, with higher mean scores indicating a greater frequency of the respective rearing behavior. The revised EMBU has demonstrated excellent reliability and validity, with a Cronbach’s α of 0.88 in the present study (28).

### Mclean Screening Instrument for Borderline Personality Disorder (MSI-BPD)

The McLean Screening Instrument for Borderline Personality Disorder (MSI–BPD), developed by Zanarini et al. in 2003, is a self–report clinical questionnaire designed to assess Borderline Personality Disorder (BPD) based on the Diagnostic Interview for DSM–IV Personality Disorders (DIPD) (29). The MSI–BPD questionnaire consists of 10 items and utilizes a self–report approach. Each item provides two response options: “Yes” and “No,” which are assigned scores of 1 point and 0 points, respectively. The total score ranges from 0 to 10 points, with a score of ≥7 indicating that the individual may meet the diagnostic criteria for borderline personality disorder (BPD). A higher score denotes more severe symptoms. Numerous studies have demonstrated that the MSI–BPD exhibits good reliability and validity (29, 30).

### Quality control

Prior to the formal survey, investigators underwent specialized training to ensure standardization and consistency in understanding, as well as to enhance their familiarity with the questionnaire. During the on–site survey, quality control inspectors conducted thorough checks on the questionnaires to verify the completeness and accuracy of the responses. Simultaneously, quality supervisors monitored the entire survey process in real time to promptly identify and address any issues. Upon completion of the survey, all collected questionnaires were subjected to a secondary review. Strict quality control measures were implemented to exclude any questionnaires that did not meet the required standards.

### Statistical analysis

Data analysis was performed using SPSS 27. Categorical data were described using proportions or rates, and intergroup comparisons were conducted using the chi–square (χ²) test. Continuous data were expressed as mean ± standard deviation, and intergroup differences were analyzed using the t–test or analysis of variance (ANOVA). Correlations between variables were assessed using Pearson correlation analysis and draw it in the relevant heat map. The NSSI (Non–Suicidal Self–Injury) scores of adolescent patients with mood disorders were set as the dependent variable, and variables showing significant differences in the Pearson analysis were selected as independent variables for multiple linear regression analysis.

## Results

### Demographics

The study comprised 101 adolescents (22.8% male, 77.2% female), predominantly junior/senior high school students (94.1%). Over half (55.4%) reported school bullying exposure, with elevated suicidality rates: ideation (85.1%), planning (67.3%), and attempts (69.3%).

### Comparison of general demographic data between two groups of adolescents with non–suicidal self–injury behavior

Among the surveyed participants, significant differences were observed between the two groups with non–suicidal self–injury (NSSI) behaviors in terms of gender, experience of school bullying, serious consideration of suicide or killing oneself, detailed planning for suicide, and actual suicide attempts in their lifetime. A gender difference was noted in the prevalence of NSSI, with females significantly outnumbering males (P < 0.01). The frequent NSSI group had a higher proportion of individuals who experienced school bullying, with statistical significance (P < 0.05). Additionally, the frequent NSSI group showed significantly higher proportions of individuals who had seriously considered suicide or killing themselves, made detailed plans for suicide, and actually attempted suicide compared to the occasional NSSI group, with statistically significant differences (P < 0.01). See **Table 1** for details.

**Table 1:** Comparison of general demographic data between two groups of NNSI behavior adolescents.

		Non–frequent NSSI group (n=49)	Frequent NSSI (n=52)	X^2^	P
Gender (%)	Male	17 (34.7)	6 (11.5)	7.691	0.006^**^
	Female	32 (65.3)	46 (88.5)		
Age (%)	<13	5 (10.2)	3 (5.8)	5.705	0.058
	13–16	29 (59.2)	42 (80.8)		
	>16	15 (30.6)	7 (13.4)		
Grade (%)	Primary school and below	3 (6.1)	3 (5.8)	2.956	0.228
	Junior high school	23 (46.9)	33 (63.5)		
	Senior high school	23 (47.0)	16 (30.7)		
	College and above	0 (0.0)	0 (0.0)		
Accommodation (%)	Yes	12 (24.5)	21 (40.4)	2.897	0.089
	No	37 (75.5)	31 (59.6)		
Long–term residence (%)	Urban	33 (67.3)	31 (59.6)	0.650	0.420
	Rural	16 (32.7)	21 (40.4)		
Only child (%)	Yes	23 (46.9)	25 (48.1)	0.013	0.909
	No	26 (53.1)	27 (51.9)		
Self–rated academic performance (%)	Excellent	5 (10.2)	6 (11.5)	5.537	0.136
	Good	18 (36.7)	9 (17.3)		
	Moderate	18 (36.7)	22 (42.3)		
	Poor	8 (16.4)	15 (28.9)		
Satisfaction with academic performance (%)	Yes	13 (26.5)	14 (26.9)	0.002	0.964
	No	36 (73.5)	38 (73.1)		
Involvement in school bullying (%)	Yes	6 (12.2)	7 (13.5)	0.033	0.855
	No	43 (87.8)	45 (86.5)		
Experience of school bullying (%)	Yes	21 (42.9)	35 (67.3)	6.105	0.013^*^
	No	28 (57.1)	17 (32.7)		
Number of friends (%)	0	11 (22.4)	5 (9.6)	3.280	0.350
	1–2	12 (44.9)	27 (51.9)		
	3–	15 (30.6)	18 (34.6)		
	6 or more	1 (2.1)	2 (3.9)		
Attitude towards physical appearance (%)	Satisfied	16 (32.6)	13 (25.0)	0.722	0.396
	Dissatisfied	33 (67.4)	39 (75.0)		
Family structure (%)	Nuclear family	24 (49.0)	30 (57.7)	3.449	0.327
	Extended family	10 (20.4)	4 (7.7)		
	Joint family	3 (6.1)	4 (7.7)		
	Other family structures	12 (24.5)	14 (26.9)		
Monthly family income (%)	Below 5000 RMB	13 (26.5)	21 (40.4)	2.986	0.225
	5000–10000 RMB	23 (46.9)	23 (44.2)		
	Above 10000 RMB	13 (26.6)	8 (15.4)		
Impact of COVID–19 (%)	Increased NSSI frequency	16 (32.7)	16 (30.8)	0.487	0.784
	No impact on NSSI	31 (63.3)	35 (67.3)		
	Decreased NSSI frequency	2 (4.0)	1 (1.9)		
Whether seriously considered suicide or self–harm (%)	Yes	34 (69.4)	52 (100.0)	18.695	0.000^**^
	No	15 (30.6)	0 (0.0)		
Whether made detailed plans for suicide (%)	Yes	23 (46.9)	45 (86.5)	17.984	0.000^**^
	No	26 (53.1)	7 (13.5)		
Whether attempted suicide in life (%)	Yes	24 (49.0)	46 (88.5)	18.487	0.000^**^
	No	25 (51.0)	6 (11.5)		

*p<0.05, ** p<0.01.

### Comparison of parenting styles and borderline personality scores among different NSSI behavior groups

The comparative analysis demonstrated that adolescents with frequent NSSI showed significantly higher scores than the non–frequent group in paternal rejection and denial, maternal overprotection and interference, and total borderline personality traits (all P<0.01, Cohen’s d>0.2). Additionally, the frequent NSSI group exhibited greater paternal overprotection and maternal rejection and denial compared to controls (both P<0.05, Cohen’s d>0.2), as presented in **Table 2**.

**Table 2:** Comparison of scores for parental rearing styles, borderline personality, and childhood maltreatment of two NSSI groups.

NSSI	Non–frequent	Frequent	t	P	Cohen d
n	49	52			
Father’s Emotional Warmth and Understanding	42.31 ± 9.439	38.81 ± 9.454	1.806	0.066	0.37
Father’s Severe Punishment	21.82 ± 7.449	25.10 ± 9.308	–1.948	0.054	–0.388
Father’s Overinvolvement	20.94 ± 4.657	22.23 ± 5.575	–1.26	0.211	–0.251
Father’s Rejection and Denial	11.61 ± 3.690	14.17 ± 4.458	–3.134	0.002**	–0.624
Father’s Overprotection	12.69 ± 3.507	14.63 ± 4.087	–2.554	0.012*	–0.508
Father’s Favoritism Towards the Subject	10.16 ± 3.249	9.44 ± 3.398	1.089	0.279	0.217
Mother’s Emotional Warmth and Understanding	43.90 ± 10.898	42.50 ± 10.748	0.649	0.518	0.129
Mother’s Overprotection and Interference	36.80 ± 7.903	42.77 ± 9.725	–3.375	0.001**	–0.672
Mother’s Rejection and Denial	15.67 ± 5.886	18.58 ± 6.341	–2.381	0.019*	–0.474
Mother’s Severe Punishment	15.65 ± 5.957	18.17 ± 7.275	–1.898	0.061	–0.378
Mother’s Favoritism Towards the Subject	10.16 ± 2.882	10.08 ± 3.136	0.144	0.886	0.029
Total Score for Borderline Personality	6.04 ± 2.622	7.73 ± 2.529	–3.297	0.001**	–0.656

*p<0.05, ** p<0.01

### Results of the correlation analysis among parental rearing styles, borderline personality traits, and non–suicidal self–injury

**Figure 1:**
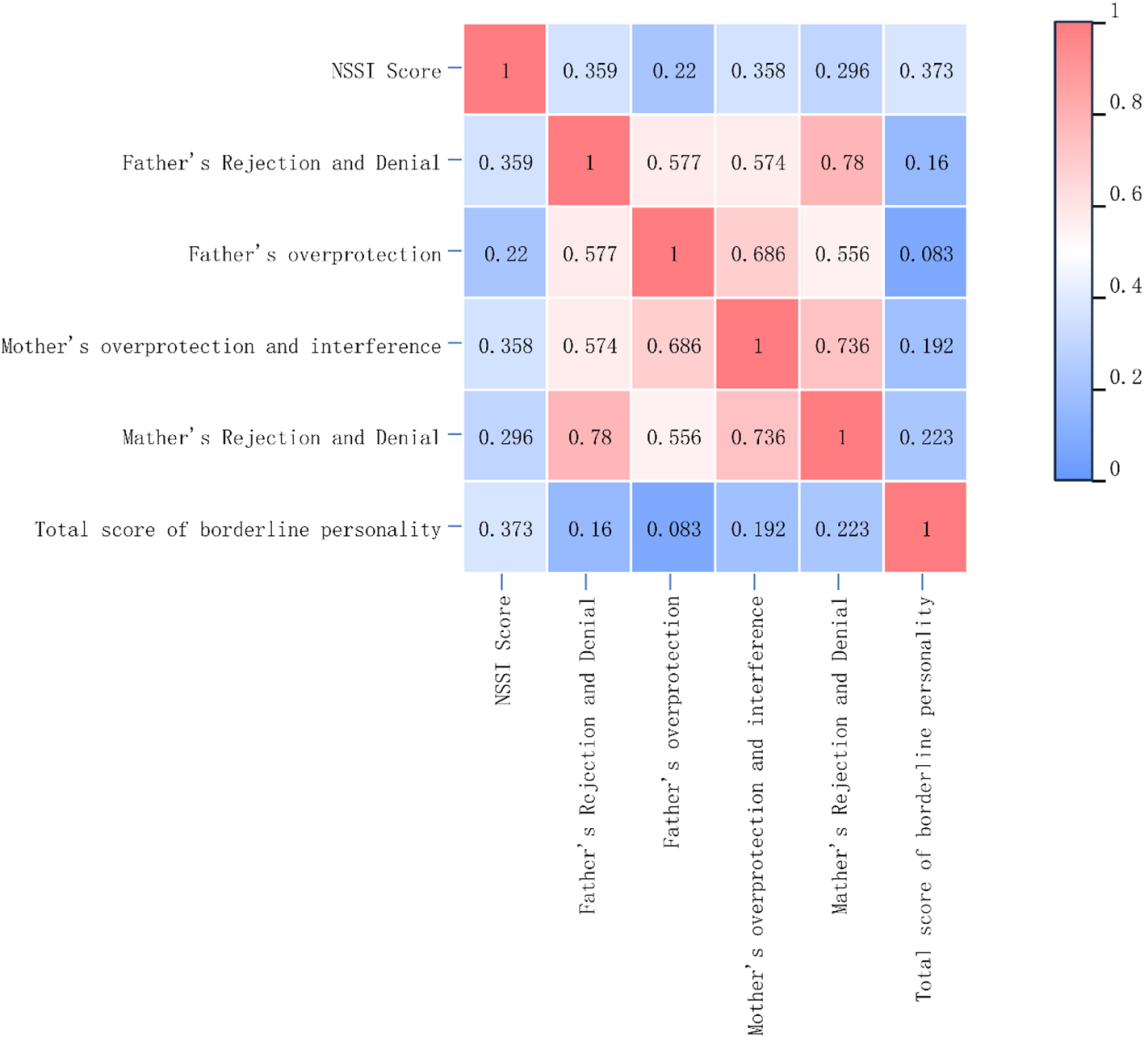
Heatmap correlation analysis results between parental rearing styles and NSSI.

Pearson correlation analysis of variables showing significant differences in t–tests with NSSI scores demonstrated significant positive correlations between NSSI scores and paternal rejection and denial (P<0.01), maternal overprotection and interference (P<0.01), maternal rejection and denial (P<0.01), and total borderline personality traits (P<0.01), as well as a positive correlation with paternal overprotection (P<0.05). These results reflect statistical associations only, and due to study limitations (e.g., unadjusted confounders, lack of temporal evidence), cannot establish that parenting styles directly cause NSSI. The correlation heatmap uses a color gradient to visualize association strength and direction: warm colors (e.g., red) indicate strong positive correlations (r>0.7), cool colors (e.g., blue) represent weak or non–significant correlations (r<0.3), and neutral colors (e.g., white) indicate moderate correlations (r≈0.5). The color scale is fixed at (0, 1) with continuous gradients to show correlation strength variations. For precise interpretation, refer to the annotated values in **Figure 1**.

### Multivariate linear regression analysis of factors influencing non–suicidal self–injury

A multivariate linear regression analysis was conducted with NSSI (Non–Suicidal Self–Injury) scores of adolescent patients with mood disorders as the dependent variable and variables showing statistically significant differences in the Pearson correlation analysis as independent variables. The results indicated that paternal rejection/denial, maternal overprotection and interference, and borderline personality traits were independent risk factors for NSSI behaviors (P < 0.05). For detailed results, refer to **Table 3**.

**Table 3:** Multivariate linear regression analysis of factors influencing NSSI.

Model	Non standardized coefficient B	t	P	95.0% confidence interval of B		VIF
				lower limit	upper limit	
(constant)	–7.176	–1.662	0.1	–15.749	1.398	
Father’s Rejection and Denial	0.881	2.613	0.01*	0.211	1.55	2.843
Father’s Overprotection	–0.318	–0.996	0.322	–0.951	0.316	2.136
Mother’s Overprotection and Interference	0.381	2.411	0.018*	0.067	0.694	2.971
Mother’s Rejection and Denial	–0.422	–1.577	0.118	–0.953	0.109	3.841
Total Score of Borderline Personality	1.135	3.475	0.001**	0.487	1.784	1.061

R^2^ = 0.276

*p<0.05, ** p<0.01

## Discussion

In contemporary society, non–suicidal self–injury (NSSI) behaviors among adolescents have garnered increasing attention from researchers. Studies have revealed that NSSI behaviors are not influenced by a single factor but rather arise from the interplay of multiple dimensions, including neurobiological, environmental, and individual psychological factors. This study explores the correlations between NSSI behaviors and various influencing factors among adolescent patients with depressive episode.

This study reveals that both the occasional NSSI group and the frequent NSSI group exhibit a higher prevalence of females compared to males, which aligns with relevant international reports indicating that the incidence of NSSI among female adolescents is 1.5 to 3 times higher than that among male adolescents (31). Females are more prone to engage in NSSI behaviors compared to males, potentially due to gender differences in coping mechanisms for stress and negative emotions (32). Research suggests that males tend to employ strategic methods to address challenges, whereas females are more likely to adopt emotional regulation as a coping strategy. However, women are more likely to fall into negative emotions such as anxiety and depression, which will increase the risk of NSSI behavior, because the effect of emotional regulation is generally not as good as that of men (33).

This study demonstrates that the frequent NSSI group exhibits a higher prevalence of experiencing school bullying, which is consistent with previous research indicating a positive correlation between bullying victimization and the frequency of NSSI (34, 35). Bullying is identified as a core risk factor for NSSI (36). Compared to peers not involved in bullying, students who experience bullying are more susceptible to NSSI and psychological symptoms (37). Research suggests that individuals who have been bullied may engage in NSSI behaviors as a means of seeking help, self–punishment, or stress relief (38). For adolescents, being subjected to bullying represents a significant source of stress, often leading to difficulties in school or social adaptation, thereby increasing the risk of engaging in NSSI behaviors (39).

The present study found that the frequent NSSI group exhibited significantly higher proportions of seriously considering suicide, formulating detailed suicide plans, and actually attempting suicide compared to the occasional NSSI group. This finding aligns with previous research (40), indicating that frequent NSSI behaviors among adolescents are often associated with suicidal ideation, suicide attempts, and actual suicidal behaviors.

This study reveals that paternal overprotection, paternal rejection and denial, maternal overprotection and interference, maternal rejection and denial are positively correlated with NSSI scores. Further research identifies paternal rejection and denial, as well as maternal overprotection and interference, as independent risk factors for NSSI behaviors. Baetens et al. (41) found a correlation between NSSI behaviors and parenting practices in community studies. This underscores the significant impact of family parenting styles on adolescents’ non–suicidal self–injurious behaviors. Parental overprotection may lead to poorer resilience in children, making them more likely to adopt passive and avoidant coping strategies when facing challenges. Paternal rejection and denial can result in lower self–esteem in children, making them more prone to self–harm when subjected to emotional attacks. Research indicates that adolescent self–injurers have lower self–esteem compared to non–self–injurers (42). Individuals with low self–esteem are more likely to experience negative emotions such as self–loathing, self–deprecation, and shame, and may believe they deserve punishment, thereby increasing the risk of NSSI behaviors. Furthermore, studies show that among adolescents with major depressive disorder (MDD), self–esteem is the most significant factor associated with NSSI, regardless of whether they are only children or not (43). Self–esteem plays a crucial role in preventing NSSI (44). Inappropriate parenting styles expose adolescents to more negative emotions, which can impair the development of their social skills and emotional regulation abilities. To alleviate emotional and psychological stress, individuals may resort to non–suicidal self–injurious behaviors. Such behaviors may serve as a means to vent emotions or seek relief, particularly when negative emotions are intense, leading individuals to adopt self–harm as a coping mechanism. These findings suggest that improving parenting styles and adopting positive parenting practices are effective strategies for preventing NSSI behaviors.

This study also found that the borderline personality score was significantly higher in the frequent NSSI group compared to the occasional NSSI group, with the difference being statistically significant. The NSSI score exhibited a clear positive correlation with borderline personality traits, and borderline personality was identified as an independent risk factor for NSSI behaviors. Borderline Personality Disorder (BPD) is a severe mental disorder characterized by emotional instability, chaotic interpersonal relationships, and a distorted self–image or sense of identity (45). Previous research has shown that adolescents with BPD exhibit a high prevalence of non–suicidal self–injury (NSSI), and NSSI is common in the early course of BPD (46). Borderline personality traits are significant predictors of NSSI behaviors (17). Individuals with BPD often display uncontrollable impulsive behaviors, poor self–regulation, and markedly impaired impulse control, making them more prone to self–harming behaviors. Additionally, BPD patients typically experience more life stressors and daily hassles than healthy individuals, and they face challenges in emotional regulation and interpersonal relationships, thereby increasing the risk of engaging in some form of self–harm among adolescents (47). Depressed youths who experience more negative life events are more likely to engage in NSSI, and negative life events indirectly influence non–suicidal self–injury through borderline personality traits (48). Among adolescent populations, self–harming behaviors often coexist with broader internalizing and externalizing problems (49) and are highly associated with Borderline Personality Disorder (BPD). Some argue that BPD traits precede and interact with self–harm (50).

This study found that paternal rejection and denial, as well as maternal overprotection, were independent risk factors for non – suicidal self – injury (NSSI). The underlying mechanisms could be further elucidated through the lens of emotion regulation theory. Paternal rejection and denial undermine adolescents’ motivation for emotion regulation by invalidating their emotions. Individuals with borderline personality disorder (BPD) traits, due to their heightened emotional sensitivity, exhibit more intense reactions to such emotional invalidation, ultimately relying on NSSI as a means of emotional anesthesia (51). On the other hand, maternal overprotection impedes the development of adolescents’ independent emotion – regulation abilities through fostering emotional dependence. This creates a synergistic effect with the anti – regulatory conflicts in individuals with BPD traits (52). Clinical interventions should target both parents’ emotion – socialization patterns and adolescents’ deficits in emotion – regulation strategies. For instance, emotion coaching training can be employed to reduce emotional invalidation, and Dialectical Behavior Therapy (DBT) skills training can be used to enhance alternative coping strategies (53).

## Conclusion

In summary, parental rearing styles and personality formation are closely related to non–suicidal self–injury behavior in adolescents. To reduce the risk of non–suicidal self–injury (NSSI) in adolescents, parents should adopt more positive parenting approaches, avoiding excessive interference, harsh denial, and striving to maintain emotional stability. “Positive parenting style” (54) refers to parents providing warm responses (timely attention, expressing care), engaging in positive communication (listening, encouraging), setting clear and consistent rules, employing guidance strategies (explaining reasons, offering choices, praising good behavior), and promoting children’s autonomy. Family interventions involve professionals providing individualized guidance, including skill demonstration, parent practice, and feedback, to assist parents in mastering and applying these approaches (55). They should also moderately educate themselves on parenting knowledge to help their children develop a healthy personality. Simultaneously, families should create a warm and harmonious environment to foster the healthy growth of adolescents’ personalities.

## Limitation

However, this study has limitations. The research subjects were limited to hospitalized adolescents aged 13–18 with depressive episode, and the sample size was relatively small, which may introduce bias in the data analysis. The sample in this study exhibited uneven gender distribution (males: 22.8%, females: 77.2%), consistent with the epidemiological characteristics of depressive disorders in adolescents, wherein the incidence of depression is notably higher among female adolescents than males (56). This imbalance may reflect the true disease distribution but requires cautious evaluation of its impact on the generalizability of the findings. Future research could specifically expand the sample of depressed male adolescents to verify the influence of parenting styles and borderline traits on non–suicidal self–injury (NSSI) in males. Conducted within the Chinese context, this study necessitates particular attention to the moderating role of cultural factors on the relationship between parenting styles and NSSI. Chinese parents generally face immense pressure regarding academic achievement, which may reinforce controlling/harsh parenting practices (e.g., excessive interference, emotional neglect), exacerbating emotional dysregulation and borderline personality tendencies in adolescents, thereby increasing the risk of NSSI (57). Simultaneously, norms of emotional restraint may hinder the identification and communication of distress between parents and children, the stigmatization of psychological problems and NSSI further conceals the issues and delays intervention (58). Future interventions should integrate these cultural factors, such as designing parent psychoeducation modules that reduce sensitivity to academic pressure, promote emotional expression, and challenge stigma.

This study did not account for other mental health conditions, such as anxiety disorders, that may coexist with depressive episode and influence non–suicidal self–injury (NSSI). Future work could systematically evaluate comorbid factors to render the model more comprehensive. Although this study identified significant associations between parental parenting styles, borderline personality, and NSSI, it is essential to underscore the fundamental limitations of the cross–sectional design: the impact of uncertainty regarding temporal sequence, systematic bias from unmeasured confounders, and the failure to capture dynamic processes. Consequently, it is impossible to establish multiple causal relationships between parental parenting styles, borderline personality, and NSSI behaviors or to analyze mediating effects among variables. The data obtained were derived from participants’ self–reports, which may be subject to recall bias regarding early childhood experiences and the influence of stigma on the results. Future research should incorporate longitudinal studies and consider other potential influencing factors to further refine the hypothetical model.

## Data Availability

The raw data supporting the conclusions of this article will be made available by the authors, without undue reservation.
